# A Curious Case of Coronary Vasospasm with Cardiogenic Shock: Type 1 Kounis Syndrome Complicated by Eosinophilic Myocarditis

**DOI:** 10.7759/cureus.4522

**Published:** 2019-04-22

**Authors:** Venkatesh Ravi, Muhammad Talha Ayub, Tisha Suboc, Tareq Alyousef, Javier Gomez

**Affiliations:** 1 Cardiology, John H Stroger Jr. Hospital of Cook County, Chicago, USA; 2 Internal Medicine, John H Stroger Jr. Hospital of Cook County, Chicago, USA; 3 Cardiology, Rush University Medical Center, Chicago, USA

**Keywords:** kounis syndrome, eosinophilic myocarditis, vasospasm, sudden cardiac arrest, cardiac magnetic resonance imaging

## Abstract

Kounis syndrome is a rare but life-threatening form of coronary vasospasm, defined by the co-occurrence of acute coronary syndrome and hypersensitivity reaction. We present a case of refractory coronary vasospasm with aborted sudden cardiac arrest secondary to type 1 Kounis syndrome, which was complicated by eosinophilic myocarditis and cardiogenic shock. A 29-year-old Hispanic woman with history of vasospastic angina, presented with recurrent episodes of angina at rest. Initial evaluation revealed hyper-eosinophilia, elevated troponin and diffuse ST segment depression on electrocardiogram (ECG). Suddenly, she developed bradycardia and had a sudden cardiac arrest. An urgent coronary angiogram after resuscitation revealed severe multifocal vasospasm which resolved following high doses of intracoronary vasodilators. Type 1 Kounis syndrome was suspected and she was initiated on intravenous corticosteroids and anti-histamines. Subsequently, she developed cardiogenic shock, and a cardiac magnetic resonance imaging (cMRI) showed diffuse subendocardial late gadolinium enhancement (LGE) suggestive of eosinophilic myocarditis. She was diagnosed with type 1 Kounis syndrome associated with eosinophilic myocarditis. Kounis syndrome should be suspected in patients with refractory vasospastic angina. When indicated, coronary angiography should be performed with administration of intracoronary vasodilators for diagnostic and therapeutic purposes. Although, definite diagnosis of eosinophilic myocarditis requires endomyocardial biopsy, cMRI can be a crucial non-invasive method for establishing the diagnosis.

## Introduction

Vasospastic angina was initially described as a clinical syndrome in 1960 and is usually associated with a low mortality and morbidity; however, it can be severe and life-threatening in rare cases, with an incidence of recurrent angina, myocardial infarction and sudden cardiac death reported at 19%, 6.5% and 3.6%, respectively [1,2]. In some series, the incidence of cardiac death has been reported to be as high as 10% based on duration of follow-up [3,4]. Kounis syndrome is a rare etiology of coronary vasospasm and is characterized by the co-occurrence of acute coronary syndrome and hypersensitivity reaction following an allergenic exposure [5,6]. The acute coronary syndrome is manifested as coronary spasm, acute myocardial infarction or stent thrombosis constituting the three variants of Kounis syndrome [5]. Type 1 variant involves patients with normal or near normal coronaries without predisposing factors of coronary artery disease (CAD), in whom allergic triggers cause coronary artery spasm. Type 2 variant includes patients with pre-existing CAD, in whom allergic triggers can cause spasm and/or plaque rupture. Type 3 variant is a new entity, occurring in patients with pre-existing CAD and drug eluting stent placement. The hypersensitivity reaction is mediated by mast cell activation leading to macrophage, T-lymphocyte activation and release of inflammatory mediators such as histamine, proteases, chemokines, cytokines, platelet activating factor, and tumor necrosis factor [5,6]. While histamine induces coronary vasospasm, the proteases can degrade the collagen cap and cause plaque rupture. We describe a challenging case of severe coronary artery spasm leading to myocardial infarction and cardiogenic shock secondary to type 1 Kounis syndrome, that was complicated by eosinophilic myocarditis. We also highlight the extremely uncommon association of type 1 Kounis syndrome with eosinophilic myocarditis.

## Case presentation

**Figure 1:**
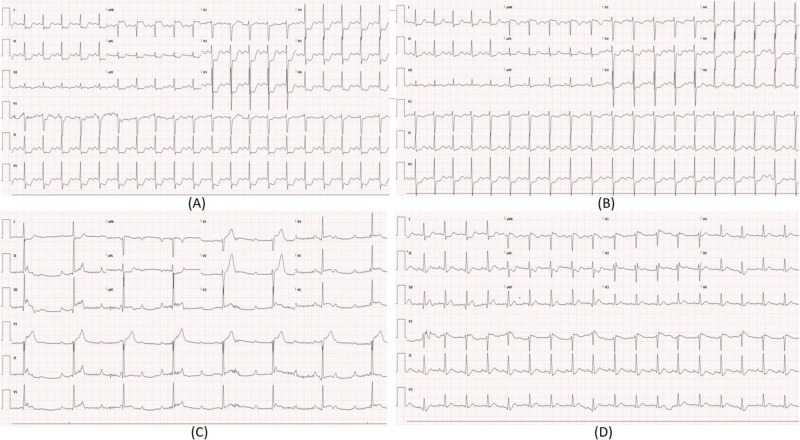
Electrocardiogram (ECG) at presentation during hospitalizations one and two. (A) ECG from hospitalization one at the time of presentation showing diffuse ST depression >1 mm, avR ST elevation >1 mm. (B) ECG from hospitalization one after nitroglycerin showing improvement in ST changes. (C) ECG from hospitalization two at the time of presentation showing ST elevation >1 mm in II, III, avF, sinus tachycardia with complete heart block and junctional escape rhythm. (D) ECG from hospitalization two following resolution of symptoms.

**Figure 2:**
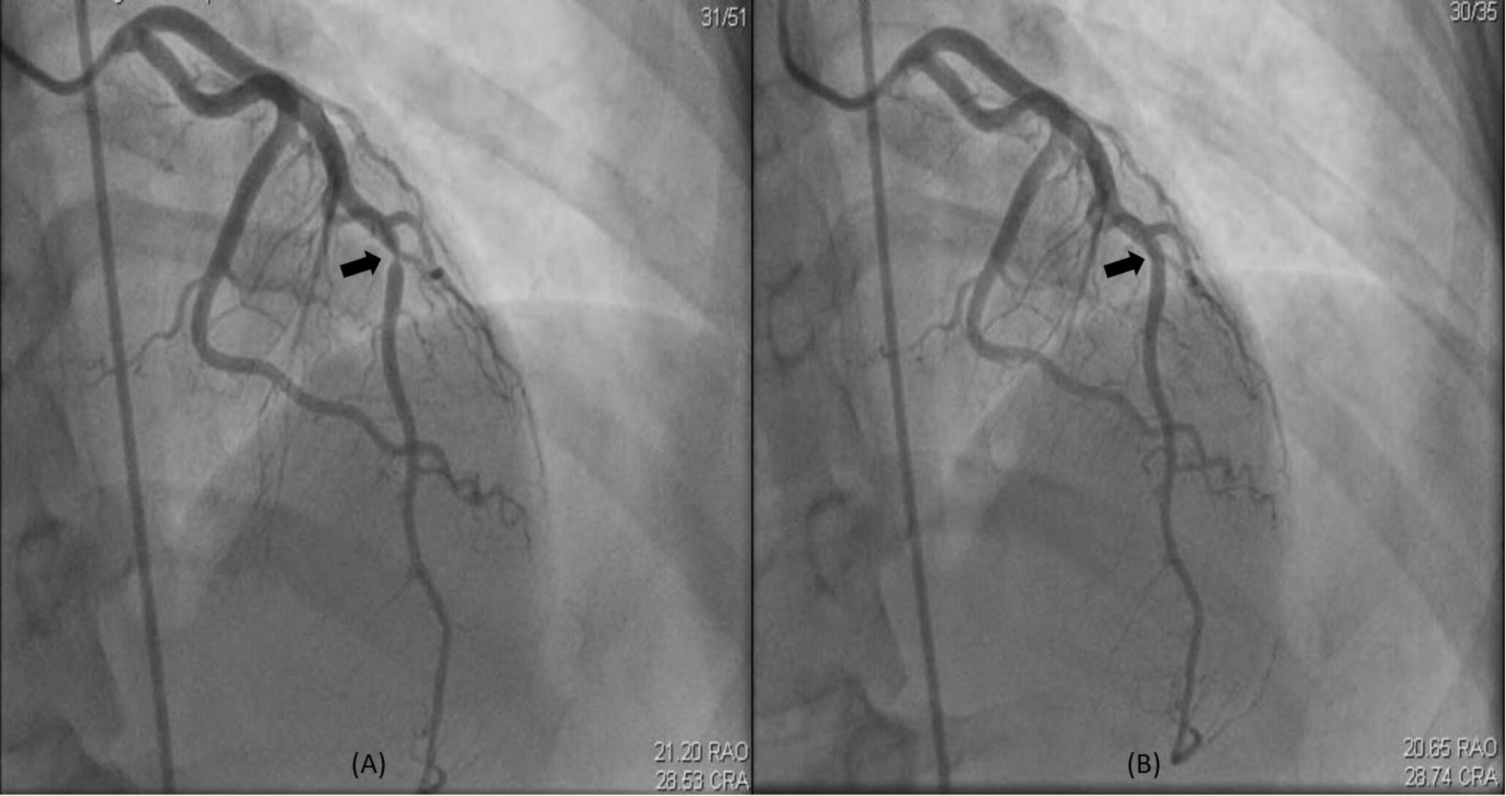
Coronary angiogram from hospitalization one. (A) Right anterior oblique – cranial view demonstrating discrete stenosis >90% in the distal left anterior descending artery after the second major diagonal branch (arrow). (B) Right anterior oblique – cranial view after intracoronary nitroglycerin showing resolution of stenosis consistent with spasm (arrow).

A 29-year-old Hispanic woman presented to our emergency department (ED) with recurrent episodes of angina at rest for four days. Each episode lasted less than five minutes and resolved spontaneously. The last episode occurred early in the morning on the day of presentation, radiated to the left arm and was associated with diaphoresis. Her past medical history was significant for asthma, allergic rhinitis, eczema and vasospastic angina for which she had been admitted to the hospital on two prior occasions. On her first hospitalization, she had a non-ST elevation myocardial infarction with diffuse ST segment depressions on electrocardiogram (ECG) (Figure 1), evidence of left anterior descending artery spasm on coronary angiogram that resolved with intracoronary nitroglycerin (Figure 2). During that hospitalization, she reported recurrence of chest discomfort with bradycardia, hypotension and went into pulseless electrical activity (PEA) cardiac arrest, from which she was successfully resuscitated and was discharged on medical therapy with amlodipine and isosorbide mononitrate. During her second hospitalization, she presented with inferior ST segment elevations associated with high-grade AV block (Figure 1), complicated by cardiogenic shock requiring vasopressors and temporary transcutaneous pacing. She recovered again with medical therapy, amlodipine was switched to diltiazem and the dose of her nitrate was up-titrated, after which she was discharged home. Transthoracic echocardiography (TTE) at the time of discharge demonstrated normal ejection fraction (EF) with no regional wall motion abnormality and she continued to do well until her current presentation. During both prior hospitalizations, her eosinophil count was elevated to >500 cells/uL (reference range [ref]: 0-400 cells/uL), while on one occasion it was >1500 cells/uL. At baseline, between the hospitalizations, her eosinophil count was normal.

**Figure 3:**
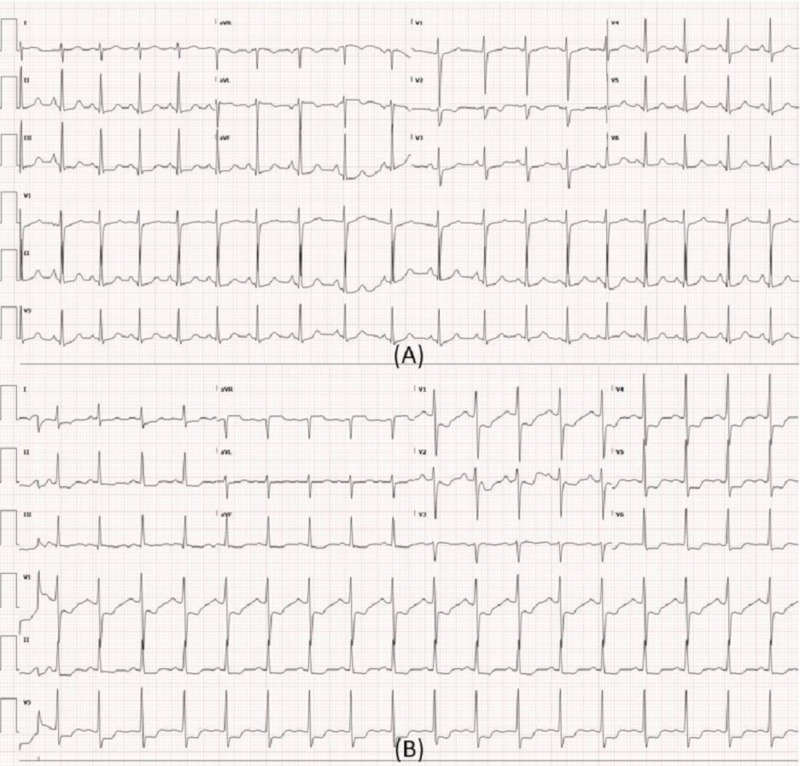
Electrocardiogram (ECG) during current hospitalization. (A) ECG at the time of presentation demonstrating sinus tachycardia with non-specific ST abnormality. (B) ECG during recurrence of chest pain demonstrating diffuse ST depression >1 mm in V1, V2, V4-V6, I, II and ST elevation in avR.

**Figure 4:**
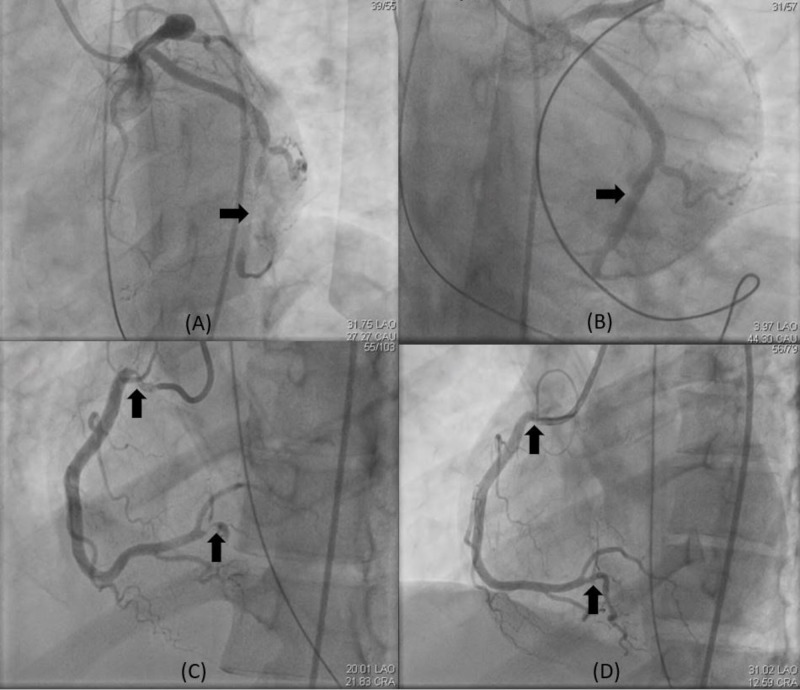
Coronary angiogram during current hospitalization. (A) Left anterior oblique-caudal view demonstrating diffuse stenosis >90% in distal left circumflex artery (arrow). (B) Antero-posterior caudal view after intracoronary nitroglycerin showing resolution of stenosis (arrow). (C) Left anterior oblique-cranial view of the right coronary artery (RCA) showing >90% stenosis in the proximal portion of RCA and proximal portion of right posterior descending artery (arrows). (D) Left anterior oblique-cranial view of the right coronary artery after intracoronary nitroglycerin and verapamil demonstrating resolution of the spasm (arrows).

**Figure 5:**
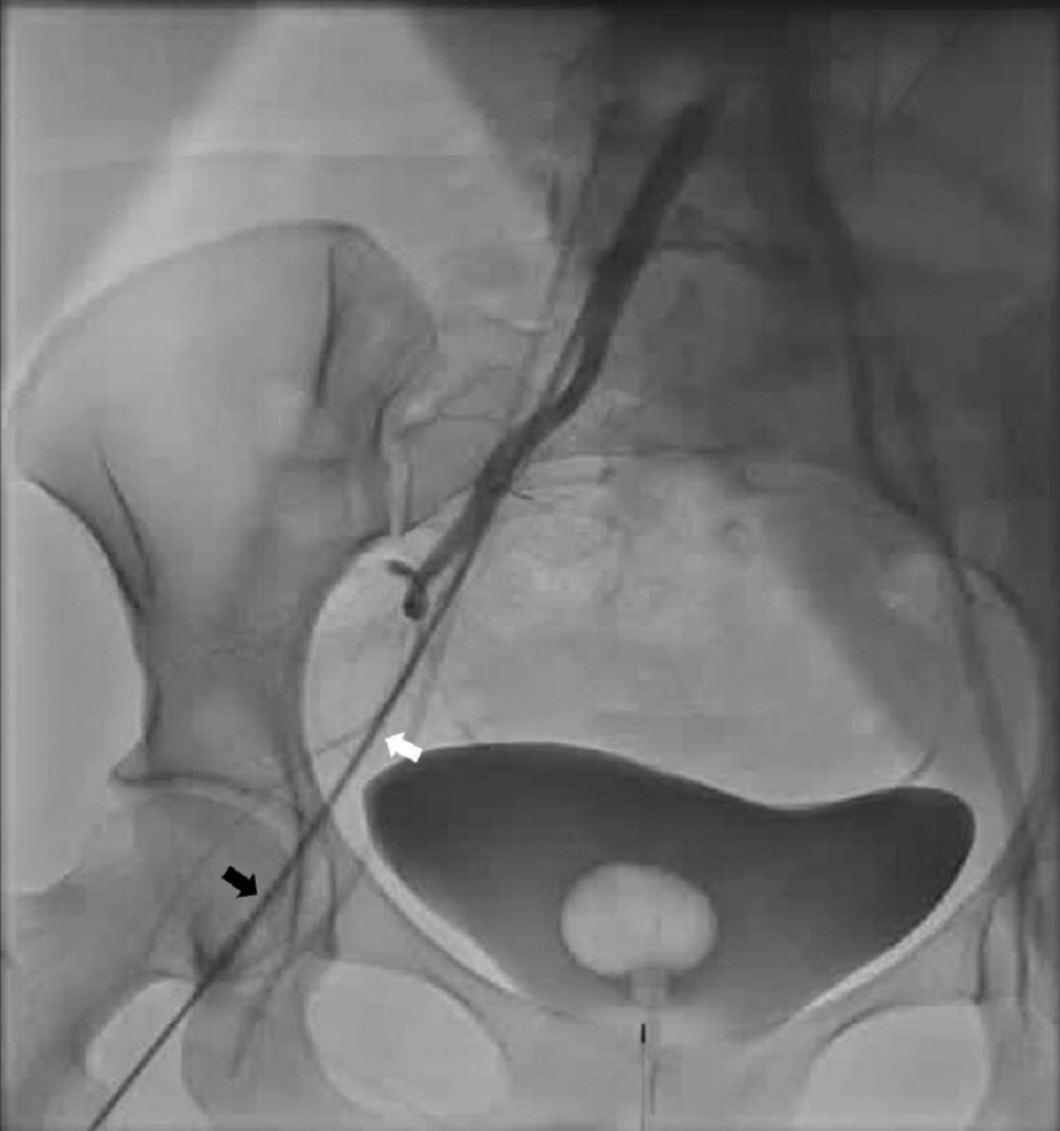
Peripheral angiogram during current hospitalization. Antero-posterior view demonstrating spasm in the right femoral artery (black arrow) and right external iliac artery (white arrow).

In the ED during current presentation, she was initially well appearing and exam was unremarkable except for tachycardia. Her vital signs were: temperature of 36.7°C, heart rate of 104 beats per minute, respiratory rate of 18 per minute, blood pressure of 98/67 mm of mercury (Hg). Her initial laboratory tests were remarkable for elevated eosinophil count of 1500 cells/uL (reference range [ref]: 0-400 cells/uL) and an elevated troponin of 0.204 ng/ml (ref: 0-0.039 ng/ml). ECG (Figure 3) demonstrated sinus tachycardia with diffuse ST segment depression <0.5 mm. Chest X-ray was unremarkable. After initial assessment in ED, she developed recurrence of chest pain and telemetry revealed complete heart block with junctional escape rhythm at a rate of 30-40 beats per minute. Repeat ECG revealed diffuse ST depression of 1 mm (Figure 3). She was initiated on transcutaneous pacing. However, her mental status worsened, she developed hypotension, hypoxemia followed by a PEA arrest. She had return of spontaneous circulation after five minutes of cardiopulmonary resuscitation. She was intubated, sedated and placed on mechanical ventilation. Intravenous nitroglycerin drip was then initiated and she was admitted to the cardiac intensive care unit. After initial response to conservative management, she again developed worsening bradycardia, hypotension and was taken for urgent coronary angiogram. There was evidence of severe diffuse spasm in the distal left circumflex artery (LCX), ostial obtuse marginal, ostial right coronary artery (RCA) and total occlusion of proximal right posterior descending artery as shown in images (Figure 4). Peripheral angiogram also revealed diffuse spasm in the right common femoral artery and right external iliac artery with sluggish flow (Figure 5). High doses of intracoronary nitroglycerin (1500 mcg) and verapamil (800 mcg) were administered. In addition, due to concern for thrombus and allergic vasospasm, intracoronary abciximab and intravenous hydrocortisone were also administered. Eventually, the coronary spasm resolved with normalization of flow (Figure 4). Due to persistent hypotension, intra-aortic balloon pump (IABP) was placed through left common femoral artery and intravenous heparin drip was initiated, following which her hypotension improved. Subsequent, TTE demonstrated severely reduced left ventricle (LV) ejection fraction (EF) of 20-25%, normal size, normal thickness, akinesis of entire inferior myocardium and reduction in right ventricular (RV) systolic function. Repeat troponin peaked at 259 ng/ml (ref: 0-0.039 ng/ml) suggesting that patient sustained infarction of large myocardial territory due to coronary spasm.

In the following hours she developed worsening tachycardia, dyspnea and oliguria. She was initiated on dobutamine drip for cardiogenic shock. A Swan-Ganz pulmonary artery catheter was placed which revealed cardiac index of 1.8 L/minute/1.73 m^2^, elevated systemic vascular resistance of 2155 dyne s/cm5, elevated mean pulmonary artery pressure of 34 mm Hg, elevated pulmonary capillary wedge pressure (PCWP) of 25 mm Hg and low mixed venous oxygen saturation (mVO2) of 48%. Her history of asthma, allergic rhinitis, and eczema raised concern for allergic-mediated coronary vasospasm as the underlying etiology of her presentation. A preliminary diagnosis of type 1 Kounis syndrome with cardiogenic shock was made and she was initiated on intravenous methyl-prednisone at 125 mg followed by 60 mg every day, intravenous hydroxyzine 10 mg every four hours and intravenous famotidine 20 mg every 12 hours.

**Figure 6:**
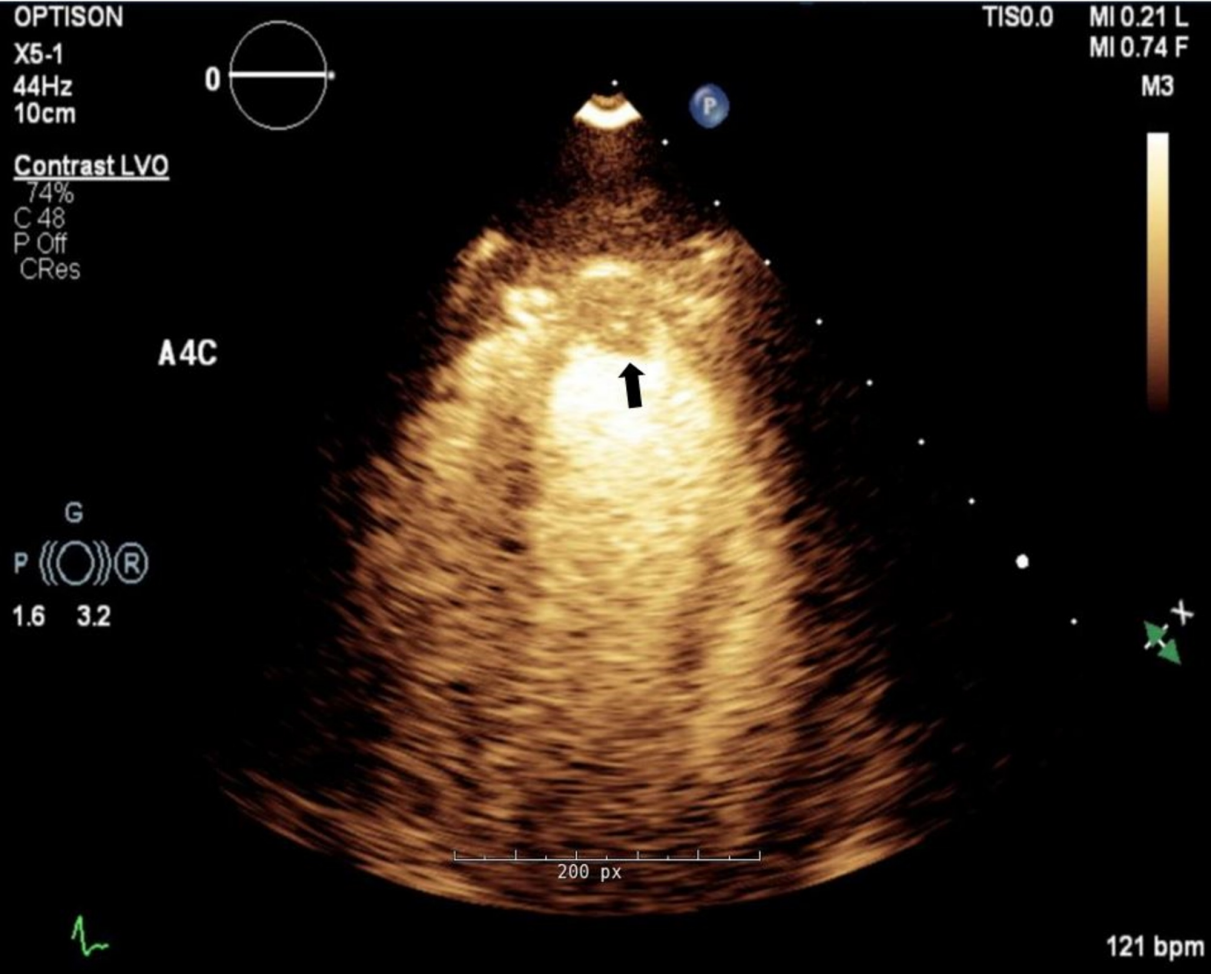
Transthoracic echocardiogram during current hospitalization. Apical four chamber view with commercial contrast demonstrating apical mass concerning for thrombus (black arrow).

**Figure 7:**
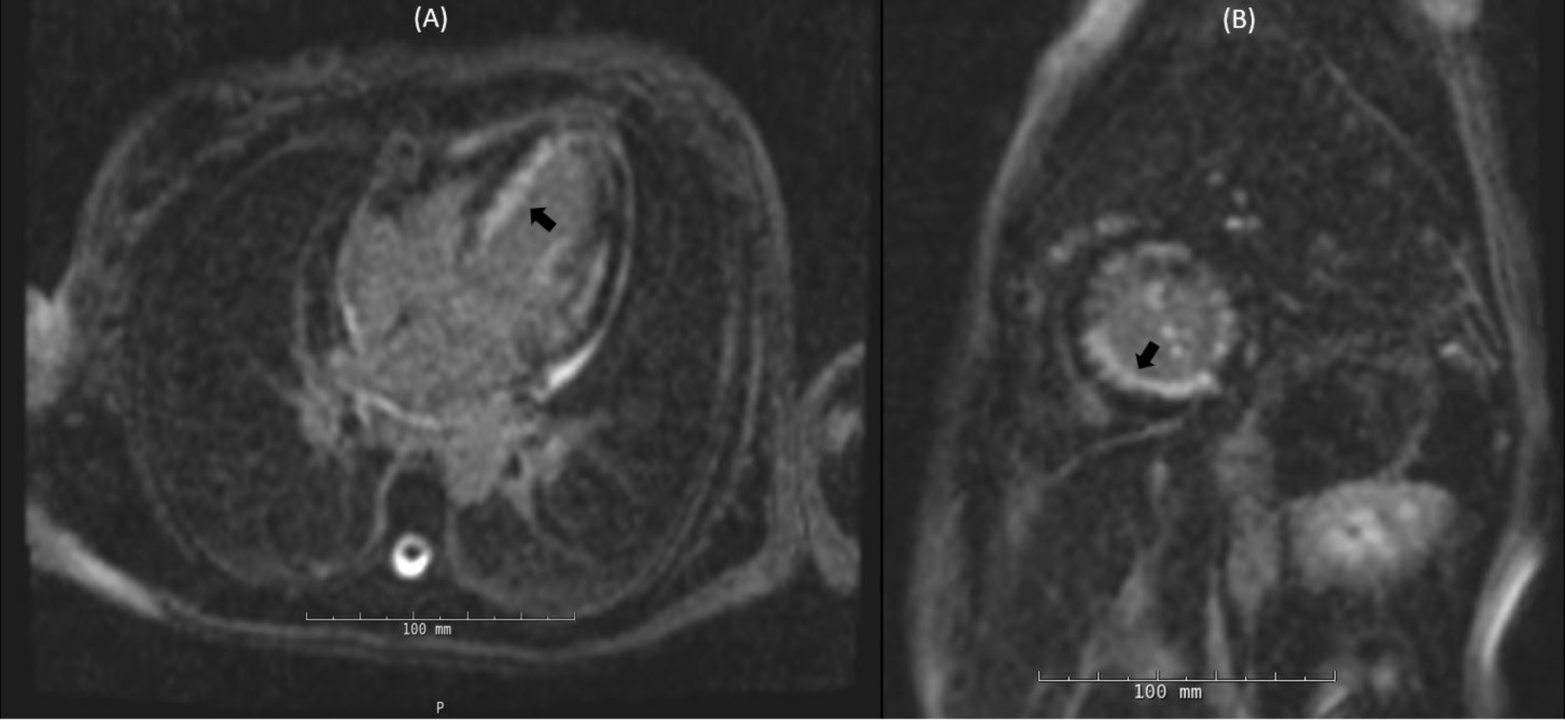
Cardiac magnetic resonance imaging during current hospitalization. Diffuse late gadolinium enhancement (black arrow) in four chamber view (A) and short axis view (B).

The inotropes and vasodilators were carefully titrated to maintain central venous pressure (CVP) of 8-12 mmHg, cardiac index of >2.1 L/minute/1.73 m^2^, systemic vascular resistance of 800-1200 dynes/cm5 and mVO2 > 65%. Repeat ECG revealed ectopic atrial tachycardia and dobutamine was switched to milrinone. She had a fever spike to 38.3°C on day two. Workup for infectious etiology was sent and broad-spectrum antibiotics were initiated. Her hemodynamics improved on supportive care with intravenous nitroglycerin, milrinone and IABP. Repeat TTE on day three showed LV EF of 15-20% and a 6 × 10 mm mass in the LV apex consistent with thrombus (Figure 6). The patient was already on therapeutic anticoagulation with intravenous heparin given IABP. Broad spectrum antibiotics were discontinued on day four after infectious workup was negative. Intravenous methylprednisolone was tapered down gradually and she was switched to oral prednisone, hydroxyzine and famotidine by day five. IABP was removed on day six and she was weaned off milrinone. Subsequent cardiac magnetic resonance imaging (cMRI) with contrast, demonstrated moderate diffuse hypokinesis of the left ventricle, normal size, normal thickness and an EF of 39%. There was diffuse subendocardial late gadolinium enhancement (LGE) of nearly the entire LV myocardium, involving approximately 50% of wall thickness (Figure 7). In addition, there was evidence of transmural LGE in the infero-lateral wall and infero-septal wall, with hypo-intensity of T1 post contrast images, consistent with acute infarct in the RCA and LCX territory. Supplemental T2 black blood sequences revealed subtle diffuse hyperintensity throughout the left ventricular myocardium, consistent with diffuse myocardial edema. A 6 × 9 mm apical LV thrombus was re-demonstrated.

Eosinophilic myocarditis was suspected given eosinophilia and cMRI findings. However, endomyocardial biopsy was deferred due to significant clinical improvement with steroids, anti-histamines, and high risk of peri-procedural complications. Her auto-immune panel was negative. Serum tryptase level was elevated to 18 ng/ml (ref: < 11.4 ng/ml) at the time of severe vasospasm which normalized following initiation on anti-histamines. Urinary N-methyl histamine level was elevated to 415 mcg/g Cr (ref: 30-200 mcg/g) and urinary prostaglandin D2 was elevated to 637 ng/L (ref: 100-280 ng/L). Her in vitro specific IgE were positive for cat, dog and mouse. Serology was negative for anti-neutrophil cytoplasmic auto-antibodies (ANCA). Workup for etiology of eosinophilia was negative for secondary parasitic infection with normal myeloid molecular profile. Her final diagnosis was refractory coronary vasospasm secondary to type 1 Kounis syndrome complicated by eosinophilic myocarditis. TTE at the time of discharge revealed an improved EF of 40%. She was discharged on day 21 with amlodipine, isosorbide dinitrate, prednisone, anti-histamines, montelukast. She continued to do well as an outpatient with repeat TTE at three months demonstrating an EF of 40%. Her prednisone dose was gradually tapered from 60 mg daily to 20 mg daily with normalization of eosinophil count.

## Discussion

We described a case of refractory coronary vasospasm with aborted sudden cardiac arrest (SCA) secondary to type 1 Kounis syndrome that was complicated by cardiogenic shock due to eosinophilic myocarditis. We highlighted our management with intracoronary vasodilators, high dose corticosteroids, anti-histamines and supportive care with IABP. Eosinophilic myocarditis was diagnosed by the diffuse late gadolinium enhancement on cardiac MRI with apical thrombus and hyper-eosinophilia. Our case is unique due to subclinical signs and symptoms of hypersensitivity at presentation, presence of systemic vasospasm and eosinophilic myocarditis complicating the clinical course.

This case highlights several diagnostic challenges. The etiology of vasospasm was initially unclear, as she was a non-smoker, with no evidence of drug abuse or other potential triggers [2,3]. Kounis syndrome, eosinophilic arteritis and small vessel vasculitis were considered as possible etiologies. Her history of atopy with asthma, allergic rhinitis and eczema raised suspicion for Kounis syndrome. The absence of symptoms and signs of hypersensitivity at presentation, does not exclude the diagnosis of Kounis syndrome, as it is not unusual for the manifestations of hypersensitivity to be subclinical [5,7]. Elevated tryptase levels and urine histamine levels which reduced to baseline following initiation of anti-histamines, were consistent with mast cell mediated vasospasm, supporting the diagnosis of type 1 Kounis syndrome [8]. Although eosinophilic granulomatosis and polyangiitis (EGPA) was a possibility given history of asthma, the absence of proven vasculitis, pulmonary infiltrates, sinusitis and negative immune panel, ANCA serology, made it unlikely [9]. Isolated eosinophilic coronary arteritis is extremely rare and was previously described in autopsy series of patients with sudden cardiac death, as a probable variant of EGPA, characterized by the isolated eosinophilic infiltration of coronary artery wall, making the diagnosis less likely [10]. Our patient also had systemic vasospasm as evidenced by common femoral artery spasm during catheter manipulation. Although common femoral artery spasm has not been reported previously, cerebral and mesenteric artery spasms have been previously documented [3]. Hence the entire arterial system appears to be vulnerable to hypersensitivity mediated vasoconstriction in Kounis syndrome [5].

The second diagnostic challenge was identifying the etiology of severely reduced systolic function and cardiogenic shock. Several probable etiologies were considered; severe diffuse ischemia with myocardial infarction, stress cardiomyopathy, fulminant viral myocarditis, giant cell myocarditis, hypersensitivity myocarditis and eosinophilic myocarditis. However, cMRI findings of diffuse subendocardial LGE and apical thrombus were suggestive of a diagnosis of eosinophilic myocarditis [11]. This was further supported by the presence of hyper-eosinophilia [12, 13]. Importantly, the presence of multifocal coronary spasm with evidence of mast cell activation indicates that the initial presentation was secondary to Kounis syndrome and argue against eosinophilic myocarditis as the solitary etiology of her presentation. Although myocardial biopsy is the definitive diagnostic modality for eosinophilic myocarditis, the risk of myocardial biopsy was deemed to outweigh any potential benefit in our patient who was improving fairly fast on the above medical regimen. Our case highlights the association of Kounis syndrome with eosinophilic myocarditis and the potential role of cMRI in establishing the diagnosis especially when the clinical suspicion is quite high.

Role of cardiac magnetic resonance imaging

We performed an extensive review of the available literature regarding cMRI findings in patients with Kounis syndrome and eosinophilic myocarditis. Contrast defect on first pass cMRI images, and the pattern of LGE were crucial in distinguishing the two diseases. Traditionally, LGE patterns on cMRI are of two types; ischemic and non-ischemic. The ischemic pattern is characterized by LGE in the subendocardial area corresponding to the territory of a culprit vessel and can extend from sub-endocardial to complete transmural involvement [12,13]. In a non-ischemic pattern, there is patchy involvement of the epicardium and mid-myocardium, but the sub-endocardium is not involved in isolation [14].

Kounis syndrome does not usually manifest with LGE on cMRI. In a study of 26 patients with chest pain, allergic symptoms and normal coronary CT angiogram, with suspected diagnosis of Kounis syndrome, none of the patients had evidence of LGE. Subendocardial contrast defect on first pass MRI images secondary to reduced perfusion from coronary stenosis and normal washout on delayed images were found to be specific findings of Kounis syndrome [14]. However, myocardial necrosis due to persistent vasospasm can result in LGE on contrast MRI images [15]. Emet et al. described MRI findings of subendocardial LGE in the hypokinetic areas and proposed that non-invasive evaluation with cMRI can be used for diagnosis of Kounis syndrome [16,17]. In a study of 137 patients with aborted SCA who underwent cMRI, LGE was demonstrated in 71% of the patients. However, the average involvement of the myocardium was about 9.9 ± 5% and diffuse subendocardial LGE was not reported. Hence, aborted SCA in our patient does not explain the diffuse subendocardial LGE [16]. Stress cardiomyopathy on rare occasions can demonstrate LGE on MRI [18]. In a study by Naruse et al. evaluating 20 patients with stress cardiomyopathy in whom cMRI was performed within one week of admission, eight patients demonstrated LGE. However, the LGE intensity (>2 SD and <5 SD) was lower than seen with myocarditis/myocardial infarction and was patchy, transmural, involving the regions with regional wall motion abnormality [19]. Hence, the pattern seen in our patient cannot be explained by Kounis syndrome or stress cardiomyopathy. Eosinophilic myocarditis has been known to demonstrate global subendocardial LGE and intraventricular thrombus during the first and second stage, respectively [20]. In a study of four patients with eosinophilic myocarditis one patient had diffuse subendocardial LGE [19]. Hence, in patients with suspected Kounis syndrome, cardiac MRI not only plays a crucial role as a non-invasive method to establish the diagnosis but also helps determine the presence of additional diagnosis like eosinophilic myocarditis.

## Conclusions

Refractory vasospasm is a rare but life-threatening emergency. Prompt coronary angiography may be considered with administration of intracoronary vasodilators for relieving vasospasm. High index of suspicion for Kounis syndrome should be maintained as early administration of corticosteroids and anti-histamines can be life-saving. cMRI is an important diagnostic for establishing diagnosis of Kounis syndrome and evaluation for potential complications. The association of eosinophilic myocarditis with Kounis syndrome should be considered, especially in patients with severely reduced systolic function and cardiogenic shock.
